# New Insights into Gastrointestinal and Pulmonary Parasitofauna of Wild Eurasian lynx (*Lynx lynx*) in the Harz Mountains of Germany

**DOI:** 10.3390/pathogens10121650

**Published:** 2021-12-20

**Authors:** Lisa Segeritz, Ole Anders, Tomma Lilli Middelhoff, Deliah Tamsyn Winterfeld, Pavlo Maksimov, Gereon Schares, Franz Josef Conraths, Anja Taubert, Carlos Hermosilla

**Affiliations:** 1 Institute of Parasitology, Faculty of Veterinary Medicine, Justus Liebig University Giessen, 35392 Giessen, Germany; Anja.Taubert@vetmed.uni-giessen.de (A.T.); Carlos.R.Hermosilla@vetmed.uni-giessen.de (C.H.); 2 Harz National Park, 38855 Wernigerode, Germany; Ole.Anders@npharz.Niedersachsen.de (O.A.); Lilli.Middelhoff@npharz.Niedersachsen.de (T.L.M.); 3 Institute of Epidemiology, Friedrich-Loeffler-Institut, Federal Research Institute for Animal Health, 17493 Greifswald-Insel Riems, Germany; Deliah.Winterfeld@fli.de (D.T.W.); Pavlo.Maksimov@fli.de (P.M.); Gereon.Schares@fli.de (G.S.); Franz.Conraths@fli.de (F.J.C.)

**Keywords:** Eurasian lynx, *Lynx lynx*, zoonotic parasites, *Aelurostrongylus abstrusus*, *Angiostrongylus* spp.

## Abstract

The Eurasian lynx (*Lynx lynx*) represents an endangered wild felid species. In Germany, it currently occurs in three isolated populations in and around the Harz Mountains, the Palatinate Forest and the Bavarian Forest. Lynx parasitic infections affect animal health and might have an influence on population performance. Therefore, we investigated the protozoan and helminth fauna of free-ranging Eurasian lynx of the Harz population with emphasis on zoonotic parasites. Individual scat samples (*n* = 24) were collected from wild animals between 2019 and 2021 in the Harz National Park and surrounding areas. In total, 15 taxa of endoparasites were detected, including seven nematodes (i.e., *Aelurostrongylus abstrusus*, *Angiostrongylus* spp., *Uncinaria stenocephala*, *Toxascaris leonina*, *Toxocara cati*, *Cylicospirura* spp. and *Capillaria* spp.), one cestode (Diphyllobothriidae) and one trematode (Heterophylidae) as well as six protozoans (i.e., *Cystoisospora rivolta*, *Cystoisospora felis*, *Toxoplasma gondii*/*Hammondia* spp., *Sarcocystis* spp., *Giardia intestinalis* and *Cryptosporidium* spp.). Moreover, first-stage larvae (L1) of spurious lungworm, *Protostrongylus pulmonalis*, originating from lagomorph preys were identified. This work represents the first report on patent *A. abstrusus* and *Angiostrongylus* spp. infections in wild German Eurasian lynxes. Some of the identified parasites represent relevant pathogens for lynxes, circulating between these carnivorous definitive hosts and a variety of mammalian and invertebrate intermediate hosts, e.g., *Sarcocystis* spp., *T. gondii/Hammondia* spp., *T. cati*, *T. leonina*, *A. abstrusus* and *Angiostrongylus* spp., while others are considered exclusively pathogenic for wild felids (e.g., *Cylicospirura* spp., *C. rivolta*, *C. felis*). This study provides insights in the occurrence of zooanthroponotically relevant metazoan (i.e., *T. cati* and *U. stenocephala*) and protozoan (i.e., *G. intestinalis*) species in free-ranging lynx. The present work should be considered as a baseline study for future monitoring surveys on endoparasites circulating in wild Eurasian lynx for appropriate management practices in lynx conservation strategies in Europe.

## 1. Introduction

**Figure 1:**
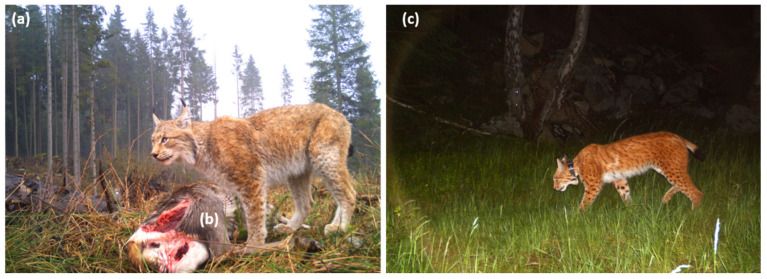
(**a**) Camera trap-images of monitored free-ranging Eurasian lynxes (*Lynx lynx*) in the Harz Mountains, Germany; (**b**) killed red deer (*Cervus elaphus)*; (**c**) GPS collar transmitter-carrying Eurasian lynx monitored within protected Harz National Park.

The Eurasian lynx (*Lynx lynx*) is the largest wild felid species in Europe. It was once widespread in Central Europe. Subsequently, it became extinct in this geographic region due to the fact of human persecution and to increased habitat losses [1]. After almost 200 years of absence, the Eurasian lynx has recently been reintroduced in some geographic regions in Germany and other central European countries, owing to several successful conservation projects [1]. Thus, this large feline carnivore species is currently strictly protected and well monitored in Germany (Figure 1). Since 2020, the Eurasian lynx has been listed as critically endangered on the Red List of the German Federal Agency for Nature Conservation [2].

In Germany, there are three isolated Eurasian lynx populations: (i) in the Bavarian Forest, (ii) in the Harz Mountains (HM) and (iii) in the Palatinate Forest [3,4]. In the monitoring year 2019/20, the HM population consisted of at least 71 independent adult lynxes and 38 juveniles [5], inhabiting an area in and around the HM (2200 km^2^), of which 247 km^2^ belonged to the Harz National Park (HNP) [1,6,7]. Eurasian lynx endoparasite fauna has been investigated in some European countries, such as Poland, Finland, Latvia, Estonia, Bosnia–Herzegovina, Slovakia, Sweden and Switzerland, where autochthonous and reintroduced lynx populations exist [8,9,10,11,12,13,14,15,16,17,18] (Table 1). Mostly, these studies focused on helminth parasites and analysed necropsied animals [9,14,19]. Consequently, reports on analyses of faecal samples in free-ranging Eurasian lynxes are still scarce [12,20]. Moreover, data on ecto- and endoparasites of wild lynx populations in Germany are still missing. The same holds true for data on the potential role of wild Eurasian lynx as apex predators in the epidemiology of zoonotic ecto- and endoparasitoses or the potential danger of parasitic transmission from wild cats and domestic/feral cats into lynx population habitats, e.g., for species of emerging and neglected feline gastropod-borne diseases of domestic pets such as aelurostrongylosis, angiostrongylosis, crenosomosis and troglostrongylosis [21,22,23] or for hookworms, where transmission from domestic cats to endangered Iberian lynx (*Lynx pardinus*) is reported [24].

Furthermore, as apex predators within a complex food chain [30], European lynxes are predisposed to acquire food-borne parasitoses (e.g., sarcocystosis, toxoplasmosis, taeniosis, mesocestoidosis, opisthorchiosis and trichinellosis), water-borne parasitoses (e.g., giardiasis and cryptosporidiosis), soil-borne parasitoses (toxocarosis and uncinariosis) and can be ecological indicators for biome health as recently reported for close related *L. pardinus* in Europe [31]. Some endoparasite infections (e.g., toxocarosis) affecting lynx health at the population level are still not fully understood and, therefore, controversially discussed [13,14]. Some ecto- and endoparasite infestations/infections can be fatal for lynx, and they represent at least additional risk factors to the fitness of these large wild felids as previously demonstrated elsewhere [32,33,34,35].

Therefore, epidemiological research on parasitic infections of wild felids in Germany, such as the Eurasian lynx and the European wildcat (*Felis silvestris silvestris*), is important for two reasons: firstly, in terms of species conservation, as parasitic infections are known to contribute to impairing lynx/wildcat populations’ health [8,14,36]; secondly, in terms of a One Health perspective, as relevant bioindicators for the ecological status of natural lynx habitats, involving many other terrestrial wild/domestic mammals, birds, amphibians, reptiles, fish, invertebrates (e.g., gastropods, insects and crustaceans), humans and plants. Thus, constant monitoring of the presence of zooanthroponotic parasitoses as well as assessment of possible transmission routes within their natural environment are highly recommended [24,31,37].

The aim of this study was thus to investigate the prevalence and diversity of endoparasites in the free-ranging HM lynx population in Central Germany, with particular emphasis on zoonotic parasitoses, by sampling living animals with non-invasive and non-disturbing methods [38,39,40], i.e., via scat sample collection from natural lynx habitats to be as close as possible to the in vivo situation.

## 2. Results

### 2.1. Endoparasite Diversity and Prevalences

**Figure 2:**
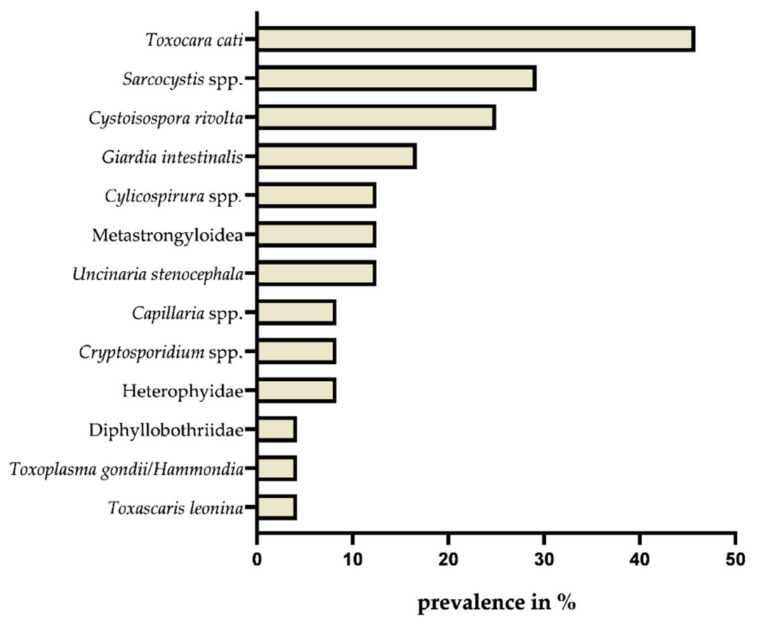
Detected helminth and protozoan endoparasite species with respective prevalence estimates, based on microscopic, *Giardia*/*Cryptosporidium*-coproantigen ELISA (ProSpecT^®^) and molecular analyses of Eurasian lynx (*Lynx lynx*) scat samples (*n* = 24) from the Harz Mountain population, Germany.

The parasitological evaluation of Eurasian lynx faeces through standardised coprological methods simultaneously unveiled a wide variety of parasite species and stages. In total 15 taxa were diagnosed including metazoan and protozoan parasites. Eight metazoan parasite genera (i.e., *Uncinaria*, *Aelurostrongylus*, *Angiostrongylus*, *Protostrongylus*, *Toxascaris*, *Toxocara*, *Cylicospirura* and *Capillaria*) belonged to the class Nematoda, one (Diphyllobothriidae) to the class Cestoda and one (Heterophyidae) to the class Trematoda. Four identified intestinal protozoan genera (i.e., *Cystoisospora*, *Toxoplasma/Hammondia*, *Sarcocystis* and *Cryptosporidium*) belonged to the phylum Alveolata (subphylum Apicomplexa) and one (*Giardia*) to the phylum Metamonada (Figure 2).

A rather large proportion, namely, 71% (17/24) of analysed lynx faecal samples contained parasitic stages (i.e., cysts, oocysts, sporocysts, eggs and larvae). The mean metazoan species richness reached 1.88/lynx. Five of the 24 analysed samples (21%) contained four or more different parasite species, indicating a wide diversity of endoparasite fauna in the examined Eurasian lynxes.

In total, 50% (12/24) of faecal samples tested microscopically positive for protozoan stages and mean protozoan species richness reached 0.79/lynx. In 54% (13/24) of the analysed lynx scat samples, nematode stages (i.e., eggs and larvae) were found and a mean nematode species richness of 0.96/lynx was calculated. Trematodes and cestodes were less frequently found with a species richness of 0.08/lynx and 0.04/lynx, respectively.

**Figure 3:**
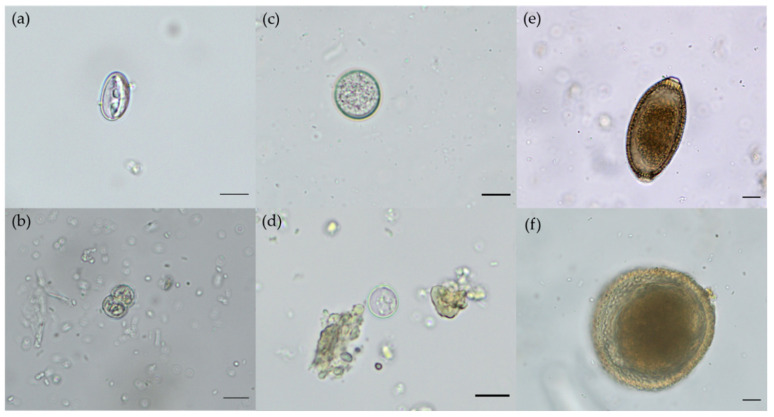
Endoparasite stages in Eurasian lynx scat samples: (**a**) *Sarcocystis* spp. oocyst; (**b**) *Sarcocystis* spp. sporocyst; (**c**) *Cystoisospora rivolta* oocyst; (**d**) *Toxoplasma*/*Hammondia* spp. oocyst; (**e**) *Capillaria* spp. egg; (**f**) *Toxocara cati* egg; scale bars: 10 µm.

Concerning helminth species richness, nematodes were the richest group with a total of seven species (i.e., *Uncinaria stenocephala*, *A. abstrusus*, *Angiostrongylus* spp., *Toxascaris leonina*, *Toxocara cati*, *Cylicospirura* spp. and *Capillaria* spp.), followed by one cestode (family Diphyllobothriidae) and one trematode species (family Heterophyidae). With regards to protozoan species richness, apicomplexans with at least five species (i.e., *T. gondii/Hammondia* spp., *Cystoisospora rivolta*, *Cystoisospora felis*, *Sarcocystis* spp. and *Cryptosporidium* spp.) were the richest group, followed by one metamonad parasite species (*Giardia intestinalis*). Overall, *T. cati* was the most prevalent parasite with eleven out of 24 (45.8%) scat samples that tested positive for this nematode, followed by the protozoan taxa *Sarcocystis* spp. and *C.* *rivolta*, with 29.2% (7/24) and 25% (6/24), respectively (for microscopic images see Figure 3).

In total, 16.7% (4/24) of the tested lynx samples were positive for *G*. *intestinalis* and in 12.5% (3/24) of the samples metastrongyloid first-stage larvae (L1) were detected.

**Figure 4:**
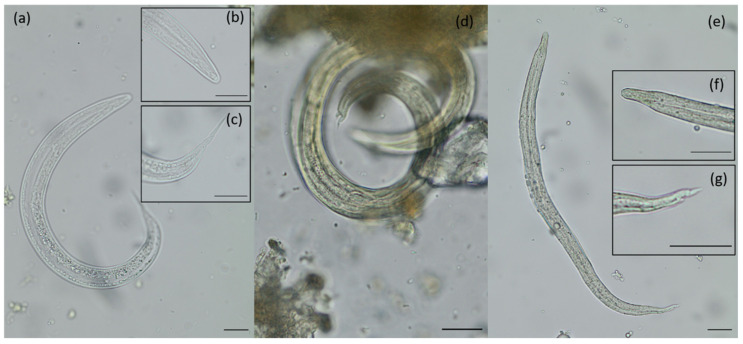
Morphological and morphometric characteristics of detected metastrongyloid first-stage larvae (L1) in Eurasian lynx scat samples: (**a**) spurious lynx parasite: *Protostrongylus pulmonalis*, 328 µm length, 21 µm width, 126 µm oesophagus length; (**b**) detail of the anterior extremity: oesophagus non-rhabditiform, 1/3–1/2 the length of the larva; (**c**) detail of the posterior extremity: long pointed and straight tail; (**d**) *Aelurostrongylus abstrusus*-L1, 388 µm length and 18 µm width, posterior extremity is characterised by a S-shaped knob-like tail with a dorsal kink; (**e**) *Angiostrongylus* spp.-like L1, 302 µm length,15 µm width, 112 µm oesophagus length; (**f**) detail of the anterior extremity: oesophagus non-rhabditiform, 1/3–1/2 the length of the larva; (**g**) detail of the posterior extremity: tip with a dorsal spine and sinus wave curve; scale bars: 20 µm.

The found larvae, belonging to the superfamily Metastrongyloidea, were identified as *A. abstrusus*-like L1, *Angiostrongylus* spp.-like L1 and a spurious lynx parasite, i.e., *P. pulmonalis* of lagomorph prey animals (see Figure 4).

Moreover, in 12.5% (3/24) of the samples, *Cylicospirura* spp. and *U. stenocephala* eggs were found. *Capillaria* spp. eggs, *Cryptosporidium* spp. oocysts, and trematode eggs were only observed in 8.3% (2/24) of the samples. The lowest prevalence, with 4.2% (1/24) was calculated for Diphyllobothriid eggs, *T. gondii*/*Hammondia* spp. oocysts and *T. leonina* eggs.

### 2.2. Molecular Analyses

#### 2.2.1. Protozoa

Two out of the four coproantigen *Giardia* spp. ELISA (ProSpecT^®^, Oxoid, Wesel, Germany) positive findings were qPCR confirmed as *G. intestinalis*. Among *Giardia* spp.-ELISA negative samples (*n* = 20), a single sample tested qPCR positive for *G. intestinalis*. Within all samples (*n* = 24), a single sample tested qPCR positive for *Cryptosporidium*. This sample was among those which had tested microscopically negative (*n* = 22). Two samples positive by microscopy tested negative by qPCR.

In six out of seven samples, microscopically positive for *Sarcocystis* spp., a nested *Sarcocystis* spp. PCR revealed amplicons of an expected size. The same was true in 14 out of 17 samples, negative during microscopy. However, in none of the *Sarcocystis* spp. positive nested PCR findings, sequencing of cloned amplicons revealed a sequence related to *Sarcocystis* spp. In one case, sequencing allowed to identify a *C. felis*-related sequence. The obtained consensus sequence can be found in the GenBank under accession number: OL689225. BLAST search revealed an identity of 99.82% to the best match with the accession number KT184364 (*C. felis* found in *F. silvestris catus*, Canada). The respective sample had tested microscopically positive for *Cystoisospora* spp.

#### 2.2.2. Nematodes

The *T. cati*-specific qPCR confirmed *n* = 5/11 samples positive by microscopy. None of the samples that tested microscopically negative were positive in the *Toxocara* spp. qPCR. No inhibitions of the qPCRs were observed.

DNA obtained from two of three microscopically lungworm larvae positive samples, could be successfully amplified by nematode specific PCRs, using the primers NC1/NC2 as reported elsewhere [41]. GenBank analyses of the obtained sequence, with the accession number MZ801778, showed a 98.87 identity in the BLAST search tool with the best match MT345056, *U. stenocephala*, found in a wild boar, France. The other sequence OK480966 showed 100% identity with the best match to KJ450994 (a sequence reported for *P. pulmonalis*, found in *Lepus europaeus*, France).

Unfortunately, none of the samples showed effective amplification on performed metastronglyloid specific qPCRs.

## 3. Discussion

The helminthofauna of the Eurasian lynx in Europe consists so far of approximately 16 nematode species, ten cestode species and one trematode species (for an overview please refer to Table 1) [9,14,22]. In line, we detected 15 taxa of parasites so far, of which five endoparasites (*Cryptosporidium* spp., *G. intestinalis*, *Sarcocystis* spp., *U. stenocephala* and *Angiostrongylus* spp.) never have been described in wild Eurasian lynx before. In related European studies, the most prevalent parasites seemed to be *T. cati* and *Taenia* spp. with the species *T. krabbei* and *T. lynciscapreoli* [9,11,13,14]. As such, the prevalence for *T. cati* infections in wild lynxes from Latvia reached up to 77% [9], in Finland and Estonia 68% [11,13], and in Switzerland 40% [8]. In the current study, in 45.8% of German lynx scat samples, *T. cati* eggs were detected, which showed similar prevalences as in other European countries. Regardless of the fact that *T. cati* is a common lynx parasite, this nematode can cause enteritis with severe clinical signs leading to cachexia and a reduced general condition in juvenile wild felids, making these young animals more receptive to other diseases [32]. There are also some reports of fatal cases of toxocarosis in juveniles [8,32]. Moreover, toxocarosis can affect humans and belongs to the most commonly reported zoonotic helminth infections worldwide, which can cause, among others, visceral larva migrans (VLM) and ocular larva migrans (OLM) syndromes [42].

In contrast to *T. cati* eggs, no *Taenia* spp. eggs were found in current copromicroscopic analyses. However, in other related studies, the methodology was mostly different, as necropsied animals were investigated and, therefore, adult *Taenia* spp. cestodes were identified. Taeniid cestodes, such as *T. krabbei* and *T. lynciscapreoli*, are known to pass their eggs mostly intermittently and inside mature proglottids into the environment. This biological feature could explain the discrepancy in the prevalences of *Taenia* spp.-infections previously reported, as the estimates may depend on the diagnostic performance of the examination method used [43]. To obtain more clarity on taeniid cestode fauna circulating in German lynxes, further studies on lynx carcasses from naturally deceased animals of the HM population, as well as analyses of lynx prey in this habitat, are highly desirable. They can also help in order to clarify another discrepancy of the current work, namely, differences between microscopic and molecular typing results. We hypothesise that the fact that we tested faecal samples with methods that vary widely in their sensitivity may explain this variance. Furthermore, lynx scat samples showed a wide endoparasite diversity with quite variable types of different parasitic stages (i.e., cysts, sporocysts, oocysts, eggs and larvae) per sample. As faeces do not represent a homogenous organic material and since we further took one portion from each investigated sample for copromicroscopic and another part for molecular analysis, the results for each method might diverge. Moreover, molecular analyses on faecal samples collected from the field proved to be challenging and possibly more specific assays need to be developed. However, despite the weakness of copromicroscopic analysis, there are still crucial advantages when compared to other parasitological diagnostic methods. One obvious advantage is, for example, the ability to detect parasite stages that other diagnostic methods (e.g., molecular analysis, muscle digestion or macroscopic pathological examination) will miss. More importantly, copromicroscopic analysis provides evidence on the patency and also detect infective stages for definitive, intermediate or paratenic hosts.

The second most frequently occurring parasitic stages microscopically diagnosed were apicomplexan *Sarcocystis* spp. oocysts/sporocysts (i.e., infective stages for various mammalian prey animals) and *C*. *rivolta* oocysts (infective stages for lynxes or other closely related wild felids (*F. silvestris silvestris*)), both well-known coccidian parasites of felines, belonging to the family Sarcocystidae. However, molecular analyses to amplify *Sarcocystis* spp. 18 S rDNA using a nested PCR resulted in amplicons of an expected size but cloning and sequencing did not reveal *Sarcocystis* sp. sequences. Thus, microscopical observations have to be regarded as non-confirmed. This zoonotic apicomplexan parasite genus is characterised by a heteroxenous life cycle, with a clear prey–predator relationship for transmission [44,45]. The fact that the carnivorous species do not only act as a final host but sometimes also as an intermediate host makes the role of the carnivore unusual [45,46,47,48]. Some *Sarcocystis* species occurring in wild animals, such as *S. nesbitti*, can be even pathogenic for humans, acting as aberrant intermediate hosts [49,50] while others are infectious for domestic and wild animals [46,50]. The typical clinical sign of sarcocystosis in predator definitive hosts is diarrhoea due to gamogony-induced enteritis after ingestion of infected intermediate hosts containing muscular *Sarcocystis*-cysts with thousands of bradyzoites [46]. In contrast, clinical signs of sarcocystosis in intermediate hosts include myositis, vasculitis, myalgia, haemorrhages, neurological signs (e.g., ataxia, paralyses), cachexia, anorexia, weight loss and abortion [44], corresponding well to clinical manifestations of *S. nesbitti*-infected human patients [49,50]. As *Sarcocystis* spp. infections led to encephalitis in Canada lynxes (*Lynx canadensis*) [51], and this parasite was the second most prevalent in the current study, we recommend investigations involving muscle and cerebral analyses of lynx, as well as prey animals (e.g., cervids, hares, mice), and molecular reprocessing of *Sarcocystis* spp.-positive samples for identification of species and for testing more alternative intermediate hosts as well as final hosts in the lynx range area within Germany.

After *Sarcocystis* spp., the second most prevalent apicomplexan parasite found in this study was *C*. *rivolta*. As for toxocarosis, cystoisosporosis was mainly observed in juvenile felids and, depending on the age, immune status and primary infective dose can lead to enteritis with catharralic diarrhoea. Unfortunately, little is known about *C. rivolta*-derived pathogenesis in young Eurasian lynxes. As stated above, in domestic cats (*F. silvestris catus*) it can cause clinical manifested cystoisosporosis with diarrhoea, dehydration and weight losses, especially in juveniles [52]. In one of the samples analysed here, the presence of *C. felis* DNA was confirmed by cloning and sequencing. Recently it was shown that *C. felis* oocysts obtained from closely related bobcats (*Lynx rufus*) in North America, can also infect domestic cats via consumption of experimentally infected mice [53]. Therefore, transmission of feline *Cystoisospora* species between free-ranging Eurasian lynxes and wild cat (*F. silvestris silvestris*) populations might also be possible to occur in the HM, but this assumption needs further clarification. Moreover, domestic cats (*F. silvestris catus*), either as feral cats or as cats kept outdoors (but owned as pets) might be identified as natural sources of highly resistant *C. rivolta* and/or *C. felis* oocysts, and perpetuating the life cycle in lynx habitats such as the HM.

Furthermore, metastrongyloid lungworm species were detected microscopically, i.e., *A. abstrusus*- and *Angiostrongylus* spp.-like larvae. These parasites are known to affect companion animals’ health status, mainly the respiratory and cardiovascular system. Molecular analysis revealed *P. pulmonalis*, which represents a lungworm-infecting lagomorphs (i.e., hares and rabbits), indicating that studies by copromicroscopy can help to reveal aspects of the trophic chain for this apex predator detecting spurious parasites of prey animals and, therefore, generating valuable data concerning wild animal diets and biome health. Beside spurious *P. pulmonalis* lungworm, so far three feline specific metastrongyloid lungworm species in European lynx populations have been described, namely, *A. abstrusus* [8,20], *Troglostrongylus brevior* [25] and recently *Crenosoma vismani* [22]. The last species (i.e., *C. vismani* (Crenosomatidae)) represents a newly described metastrongyloid nematode of the European lynx [22]. By morphological and morphometric analyses, *A. abstrusus*-like L1 were detected in collected lynx faeces. Unfortunately, the molecular analysis failed for *A*. *abstrusus*, most probably either due to the insufficient DNA quality or bacteria-mediated DNA degradation. The same holds true for observed *Angiostrongylus* spp.-like L1, where molecular analysis also failed, but classical tail morphology as well as morphometry (see Figure 4) indicated similarities to the genus *Angiostrongylus*. We hypothesise, considering the known literature of metastrongyloid nematodes infecting wild felids and morphological observations of the detected larvae, that either *T. brevior* or *Angiostrongylus chabaudi* are the most probable species circulating in the HM lynx population, which has so far been isolated from other lynx occurrences. Among them, *A. chabaudi* has not been described so far to infect the Eurasian lynx; however, it is recognised to infect European wildcats *(F. silvestris silvestris)* [54,55], an endemic feline species in the HM lynx range habitats. We also cannot exclude the possibility that observed *Angiostrongylus* spp.-like L1 belong to the morphological similar species *T. brevior* (Crenosomatidae), which is rarely reported in Germany [56]. Prevalences for lungworms in *F. silvestris silvestris* in Germany reached 17% [56] and *A. chabaudi* was molecularly detected in a German wildcat by Hirzmann et al. (2018) (GenBank accession number: MH899656). Furthermore, wildcats are suggested to be a sylvatic reservoir for feline lungworms [57,58].

New approaches for better DNA isolation/preservation of lungworm larvae obtained from freshly passed samples by Baermann funnel technique and ongoing molecular analyses will hopefully clarify species spectrum of metastrongyloid species in the near future. As metastrongyloid lungworm infections seem to spread within Europe [41,59] and South America [60,61], new species have been identified and prevalences seem to be rising, thus further research in neglected gastropod-borne diseases is valuable [22,62,63,64,65,66,67].

In line with these results, we detected not only gastropod-borne nematode and trematode (Heterophyidae) infections but also zoonotic fish-borne cestode infections (dibothriocephalosis (former diphyllobothriosis) or spirometrosis (sparganosis)). The former is an interesting finding, considering that HM lynxes were not yet observed to consume fish [30]. Pseudophyllidean infections with neglected zoonotic-relevant species such as *Dibothriocephalus latus*, *Spirometra janickii* and *Spirometra erinaceieuropae* were already described in Eurasian lynx [10,12] and, thus, demanding improved surveillance.

Furthermore, the findings of *Cylicospirura* spp. eggs in the present study add novel data on the helminth fauna of *L. lynx*. In Europe, cylicospirurosis is rarely reported in wildcats (*F. silvestris silvestris*) and the life cycle of *Cylicospirura*, which involves insects as intermediate hosts and small vertebrates as paratenic hosts, remains in many parts uncovered [68,69,70]. Pathology of neglected cylicospirurosis, which affects mainly wild felids, among them also lynxes (*L. canadensis* and *L. rufus)*, is associated with stomach nodules due to severe granuloma formation [68,71,72].

In addition to the abovementioned parasites, this study detected for the first time two protozoan genera (i.e., *Giardia* and *Cryptosporidium*) in Eurasian lynxes by coproantigen ELISAs, qPCR analyses and carbolfuchsin-stained faecal smears. As there is a lack of studies in intestinal protozoan parasite prevalence in feline wildlife [36], our results highlight that further research is necessary in this field. Evaluating the results further, it stands out, that two of the four *G. intestinalis*-positive samples were collected from lynx family groups with juveniles. In these juvenile scat samples, a much higher signal intensity in performed coproantigen ELISAs was observed, when compared to positive ones from adults. We hypothesise, that similar to other mammalian host species, juveniles are more frequently infected thereby shedding more infective *G. intestinalis* cysts into environment as compared to partially or fully immunocompetent adults thereby contributing significantly to habitat contamination. In addition, a potentially zoonotic helminth species (i.e., *U. stenocephala*) was molecularly confirmed in lynxes. In general, this hookworm species is described to infect more frequently canids, but rarely also found in felids [73,74]. To the best of our knowledge, *U. stenocephala* has not yet been described in Eurasian lynx populations in Germany. Nevertheless, some studies identified *Uncinaria* sp. infections in Eurasian lynxes in Switzerland [8] and in a closely related Canada lynx (*L. canadensis*), *U. stenocephala* was detected as well [75]. As domestic cats are reported to act as reservoir for hookworms affecting the Iberian lynx (*L. pardinus*), companion animals could play a similar role in the epidemiology of *Uncinaria* sp. in Eurasian lynx [24,35,76].

Overall, the spectrum of protozoa and helminth parasites with respective prevalences reported in this study are in accordance to previous reports on parasite fauna of free-living lynxes from other European countries, but also added new species to the list of *L. lynx* parasites [8,9,11,12,13,14]. Findings on aelurostrongylosis, angiostrongylosis, dibothriocephalosis as well as sarcocystosis and cylicospirurosis, call for more research activities, not only on definitive hosts and intermediate hosts but also on paratenic hosts to generate novel insights into the epidemiology of neglected parasitoses circulating in Eurasian lynx populations.

## 4. Materials and Methods

### 4.1. Study Area and Sampling Collection

**Figure 5:**
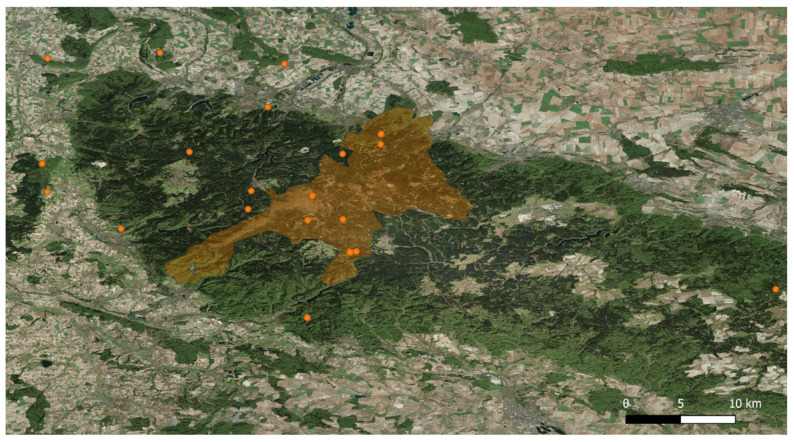
Eurasian lynx (*Lynx lynx*) faecal sample collection sites. The orange area indicates the protected Harz Mountain National Park in Germany; each dark orange dot represents a faecal collection spot. From 20 collection sites, 24 lynx faecal samples were obtained.

Scat collection was performed in the range of the Harz lynx population in Germany (see Figure 5). This range includes an area of 2200 km^2^ forested midland mountains, the HM which, in turn, contains the approximately 247 km^2^ protected area of the HNP (51°47′0″ N, 10°34′0″ E), which is part of the European nature conservation network of Natura 2000. The altitude in the HM varies from less than 300 m at the northern and southern borders, to 1141 m above sea level on the summit of the Brocken Mountain [6]. Around 75% of the HM are forested, with variable types of mainly anthropogenic spruce forest. Beech, which is actually dominant here, has been pushed back by human influence into rather small areas of the low mountain range.

Some animals living in the HM, including mammals, birds, invertebrates, amphibians, reptiles, and fish, belong to endangered species. By scat analysis, nine prey animals of lynx could be assigned to the species level. Those include roe deer, red deer, red fox, European water vole (*Arvicola terrestris*), bank vole (*Myodes glareolus*), common vole, red squirrel (*Scirus vulgaris*), brown rat (*Rattus norvegicus*) and black woodpecker (*Dryocopus martius)* [30]. However roe deer and red deer are the main prey species in the Harz lynx population [30,77]. In 2000, the reintroduction programme of *L. lynx* into the HM started. As the lynx range is wider than the National Park borders (Figure 5), these wild felids are also inhabiting surrounding areas, which are dominated by agriculture. In the western and southern foreland of the HM the forest cover reaches a maximum of about 25% whereas north and east of the area, forest is scarce due to the fertile soils allowing profitable agricultural production [33]. Beside the Eurasian lynx, there is another wild feline predator living in this area, namely, the European wildcat (*F. silvestris silvestris*).

During the general lynx monitoring of the HNP, a total of 24 faecal samples of Eurasian lynxes were collected at 20 different collection sites between 2019 and 2021. The coordinates of these collection sites were recorded, with seven locations within the HNP and 13 at surrounding areas (see Figure 5). Lynx faeces were mostly located next to killed prey, e.g., red deer (*C. elaphus)* and roe deer (*C. capreolus*) (Figure 1). In some cases, more than one scat was found at a killing site. The allocation of the faecal samples to specific lynx individuals was possible because the animals wore collar transmitters or were genetically sampled (e.g., saliva sample from a prey animal close to where the faecal sample was found). In addition, prey killing sites were often monitored with camera trap images, so that female lynxes with offspring could be identified. Collected samples were immediately fixed in 80% ethanol until further investigation. Thereafter, the scat samples were transferred to the Institute of Parasitology at Justus Liebig University Giessen (JLU), Giessen, Germany, for further analyses.

According to Council Regulation (EC) No 338/97 of 9 December 1996 on the protection of species of wild fauna and flora by regulating trade therein, faeces are defined as natural excretions and, thus, not considered as products of wild animals; therefore, no official permit was necessary to perform this copromicroscopic study. Furthermore, official employees of the HNP were exclusively involved in scat sample collections.

### 4.2. Coprological Analyses

#### 4.2.1. Microscopical Analyses

The faecal samples were copromicroscopically analysed by using the standard sodium acetate acetic acid formaldehyde (SAF) technique previously used for wild felids and domestic animals [43,78,79]. SAF stock solution was prepared with 15 g sodium acetate, 20 mL acetic acid, 40 mL formaldehyde (37%) and 925 mL tap water. Lynx faeces (4 g) was filled in a sampling tube with 8 mL SAF solution and suspended. After filtering the sample through a layer of gauze into a conical centrifuge tube, it was centrifuged at 2000 rpm for 1 min. The supernatant was discarded and 7 mL of 0.9% sodium chloride solution and 2 mL of acetic acid ethyl ester was added and stirred up. Then, a final centrifugation step followed at 600× g for 3 min, and the sediment was examined.

The light microscopy-based identification of parasitic stages such as eggs, larvae, cysts, sporocysts and oocysts was based on morphological/morphometric characteristics. Moreover, carbolfuchsin-stained faecal smears according to [80] were performed for the detection of *Cryptosporidium* spp. oocysts.

Metastrongyloid lungworm first-stage larvae (L1) were morphologically identified by body length and width, oesophagus form (non-rhabditiform), ratio of oesophagus length to body length (1:3–1:2) as well as typical tail morphology as reported by other authors [25,58,66,81] (see Figure 4).

#### 4.2.2. Coproantigen-ELISAs

Additionally, coproantigen-ELISAs (ProSpecT^®^, Oxoid, Wesel, Germany) were carried out for the detection of *C. parvum* and *G. intestinalis* antigens in faecal samples of mammalian species.

#### 4.2.3. Molecular Analyses

##### Protozoa

Faecal samples (*n* = 24), preserved in 80% ethanol, were washed two times in distilled water. DNA was extracted from the final faecal suspension (200 µL) using a commercial kit, the Quick-DNA Fecal/Soil Microbe Miniprep Kit (Zymo Research Europe, Freiburg, Germany).

The following real-time PCR was used to detect *G. intestinalis* and *Cryptosporidium* spp. using previously publishes primers and probes (Table 2).

For *G. intestinalis* we used Gd-80F 5′-GACGGCTCAGGACAACGGTT-3′, Gd-127R 5′-TTGCCAGCGGTGTCCG-3′ as primers and as a probe Gd-FT 5′-FAM-CCCGCGGCGGTCCCTGCTAG-DDQ1-3′ [82]. For *Cryptosporidium* spp. primers Cp-583F 5′-CAAATTGATACCGTTTGTCCTTCTG-3′ and Cp-733R 5′-GGCATGTCGATTCTAATTCAGCT-3′ as well as the probe Cp-TRT 5′-Texas Red-TGCCATACATTGTTGTCCTGACAAATTGAAT-DDQ2-3′ were used [82].

The qPCR reactions (25 µL final volume) consisted of 12.5 µL of QuantiTect Multiplex PCR NoROX Master Mix (Qiagen, Hilden, Germany), 3 mmol/L MgCl_2_, 0.4 µmol/L of *G. intestinalis* primers and 0.12 µmol/L of the *G. intestinalis* probe, 1 µmol/L *Cryptosporidium* spp. primers and 0.5 µmol/L of the *Cryptosporidium* spp. probe and 3 µL of a 1:5 diluted template DNA.

To monitor the inhibition of the real-time PCRs, a heterologous plasmid with DNA sequences resembling the enhanced green fluorescent protein (EGFP) gene was added to the reaction mix in all real-time PCRs. The internal control PCR included the primers EGFP1-F, EGFP2-R and the probe EGFP1 as previously reported [83].

Cycling condition for qPCR was as follows. In case of *G. intestinalis* and *Cryptosporidium* spp. the cycling conditions consisted of 15 min at 95 °C denaturation and 45 cycles of 60 s at 95 °C denaturation, 30 s at 55 °C annealing and elongation for 30 s at 72 °C with a ramping of 3.3 °C/s. The measurement of the fluorescence was taken at the end of the annealing.

*Sarcocystis* spp. 18S rDNA was detected by nested PCR using 1 µL of extracted template DNA. The external *Sarcocystis* spp.-specific PCR, amplified fragments of a size of around 900 bp using the primers SarcoFext and SarcoRext [85]. For the internal PCR, 1 µL of the external reaction was used, employing the primer pair SarcoFint/SarcoRint [85]. The external as well as the internal PCR was performed in a final reaction volume of 25 µL with 1 unit of Taq DNA polymerase per reaction (Stratec molecular, Germany) under the following conditions (final concentrations): 20 µg/mL BSA, 1 × reaction buffer supplied with the polymerase, 1.5 mM MgCl_2_, 250 µM dNTPs, 0.5 µM of each primer. The following cycler program was used: 94 °C (4 min), 40 cycles of 94 °C (40 s), 58 °C (1 min), 72 °C (1 min) and a final extension at 72 °C (5 min). Five µL of each product were examined in 1.5% agarose gels stained with ethidium bromide.

For sequencing of amplicons, bands of the expected size were excised from agarose gels and purified with a commercial kit (NucleoSpin^®^ Gel and PCR Clean-up, Macherey-Nagel, Düren, Germany), following the manufacturer’s instructions. Purified amplification products were cloned into a commercially available vector (pGEM^®^-T Easy Vector System I, Promega, Mannheim, Germany) and used to transform chemically competent *Escherichia coli* (OneShot TOP10, Thermo Fisher Scientific, Langenselbold, Germany). The transformed *E. coli* were cultivated and the plasmid DNA was subsequently purified using a commercial kit (QIAprep Spin Miniprep Kit, Qiagen, Hilden, Germany) according to the manufacturer’s instructions. Sequencing was performed using the BigDye Terminator v1.1 Cycle Seq. Kit (Thermo Fisher Scientific, Langenselbold, Germany) and passed through NucleoSEQ Columns (Macherey-Nagel, Düren, Germany) for cleaning up nucleic acids, in an ABI 3130 capillary sequencer (Thermo Fisher Scientific, Langenselbold, Germany).

The forward and reverse sequences were aligned, if necessary trimmed based on primer sequence information and the consensus sequences for the individual cloned amplification products compared to sequences stored in GenBank, EMBL, DDBJ, or RefSeq using BLASTn with standard conditions.

##### Nematodes

All samples were analysed by *Toxocara* spp. multiplex qPCR, using Tcanis-F 5′-GCGCCAATTTATGGAATGTGAT-3′ and Tcati-F 5′-ACGCGTACGTATGGAATGTGCT-3′ as forward primers and TCC-R 5′-GAGCAAACGACAGCSATTTCTT-3′ as the reverse primer [84]. As probes, for *T. canis* Tcanis-S 5′-FAM-CCATTACCACACCAGCATAGCTCACCGA-3′-BHQ1 and for *T. cati* Tcati-S 5′-Cy5-TCTTTCGCAACGTGCATTCGGTGA-3′-BHQ3 were used [84]. Including 12.5 µL of Master Mix, 0.3 µmol/L of each primer, 0.1 µmol/L of each probe and 2.5 µL of a 1:5 diluted template DNA were used. The cycling conditions consisted of 15 min at 95 °C denaturation and 45 cycles of 60 s at 94 °C denaturation and 60 s at 60 °C annealing and elongation with measurement at the end.

The samples being microscopically positive for metastrongyloid L1 (*n* = 3) were further analysed by PCR and sequencing, in order to identify them to species level. DNA was extracted using a commercial kit (QIAamp Fast DNA Stool Mini Kit^®^, Qiagen, Hilden, Germany). For the first PCR the universal nematode primers NC1 5′-ACGTCTGGTTCAGGGTTGTT-3′ and NC2 5′-TTAGTTTCTTTTCCTCCGCT-3′ [86] were used in a reaction volume of 50 μL, HOT FIREPol^®^ Blend Master Mix (Solis BioDyne, Tartu, Estonia) and 5 μL of DNA template with following conditions: denaturation 95 °C 15 min, 35 cycles of denaturation 95 °C 20 s, annealing 52 °C 30 s and extension 72 °C 30 s, followed by a final elongation of 72 °C 5 min. PCR products were analysed by gel electrophoresis and used as templates for specific nested PCRs. For identification of *A. abstrusus* and *T. brevior*, a duplex three step PCR was performed, using the forward primers TrogloF 5′-GCACTTGAAATCTTCGACA-3′, AeluroF 5′-GCATTTATGCTAGTGATATC-3′ and the single reverse primer MetR 5′-CCGCTAAATGATATGCTTA-3′ as described elsewhere [87]. The final reaction volume of 20 μL consisted of 4 μL 5 × HOT FIREPol^®^ Evagreen^®^ qPCR Mix Plus (Solis BioDyne, Tartu, Estonia), 250 nM of each primer, 13.5 mL sterile water and 1 μL template. Cycling protocol was 95 °C 15 min, 35 cycles of denaturation 95 °C 20 s, annealing 59 °C 20 s, and extension 72 °C 30 s.

As these metastrongyloid real-time PCRs were negative or inconclusive and the quantity of the amplicon-DNA from the first PCR was low, a second nested conventional PCR was performed, using the primers NC1/MetR. Two samples were purified and sent to a commercial sequencing service (LGC Genomics, Berlin, Germany). The obtained sequences were analysed by BLAST search (http://www.ncbi.nlm.nih.gov/BLAST/; accessed on 15 December 2021) and then submitted to the GenBank.

## 5. Conclusions

We investigated the endoparasite fauna of free-ranging lynxes of the Harz lynx population and report for the first time on the occurrence of feline aelurostrongylosis and angiostrongylosis. Many parasite species found in Eurasian lynx can also be found in wild cats and even in domestic cats that live in direct contact with humans. Therefore, a One Health approach is requested in order to identify parasite transmission routes. Relevant zooanthroponotic parasites, such as water-borne (i.e., *G. intestinalis*) as well as soil-borne (i.e., *T. cati* and *U. stenocephala*) infections were detected. These results may help to improve our understanding of the impact of these parasites on the health status of wild lynx and also on obligate intermediate or paratenic hosts within the range of the ecological habitat.

## Figures and Tables

**Table 1:** Reported endoparasites with prevalences in Eurasian lynx (*Lynx lynx*) including findings of present study.

Parasite	Country *	Prevalence Range in %	Prevalence Current Study in % (95%CI)	Literature
Nematodes				
*Toxocara cati*/*Toxocara* sp. **	P, B, L, F, E, G, Swi	14.7–81.8	45.8 (25.9–65.7)	[8,9,11,12,13,14,16]
*Toxascaris leonina* **	B, Swi, G	1.7–9.1	4.2 (0–12.2)	[8,14]
*Ancylostoma tubaeforme*/*Uncinaria* spp. **	P, B, F, Swi, G	0.6–18.2	12.5 (0–25.7)	[8,12,13,14]
*Aelurostrongylus abstrusus* **	P, Swi, G	8.6–21.1	4.2 (0–12.2)	[8,20]
*Troglostrongylus brevior*	L	x	0.0 (0)	[25]
*Crenosoma vismani*	L	x	0.0 (0)	[22]
*Angiostrongylus* spp.-like larvae **	G	-	4.2 (0–12.2)	Current study
*Metastrongylus* sp.	P	1	0.0 (0)	[12]
*Protostrongylus pulmonalis* **	G	-	4.2 (0–12.2)	Current study
*Capillaria aerophilus*/*Capillaria* sp. **	F, Swi, E, G, L, P	5–45.7	8.3 (0–19.3)	[8,9,11,12,13]
*Trichuris* sp.	Swi	8.6	0.0 (0)	[8]
*Capillaria felis-cati*	L	4	not determined	[9]
*Nematoda* sp.	L	3.1	not determined	[9]
*Nematodirus* sp.	P, Swi	1–1.7	0.0 (0)	[8,12]
*Trichinella* sp.	L, Swi, E	29.6–46.4	not determined	[8,9,11]
*Trichinella nativa*	L, E	3–20	not determined	[15,26]
*Trichinella britovi*	B, E, L, Swi, Sl	27.3–97	not determined	[14,15,18,26,27]
*Trichinella spiralis*	E	4.4	not determined	[15]
*Cylicospirura* spp. **	G	-	12.5 (0–25.7)	Current study
Cestodes				
*Dibothriocephalus latus*/Diphyllobothriidae **	P, F, E, G	3–5.4	4.2 (0–12.2)	[11,12,13]
*Taenia* sp.	P, F, Swi	5.2–28	0.0 (0)	[8,12,13]
*Taenia pisiformis*	L, E	100	not determined	[9,11]
*Taenia laticollis*	E	40.5	not determined	[11]
*Taenia lynciscapreoli*	B	72.7	not determined	[14]
*Taenia krabbei*	B	45.5	not determined	[14]
*Taenia taeniaeformis*	E	2.7	not determined	[11]
*Taenia hydatigena*	E	2.7	not determined	[11]
*Mesocestoides* sp.	F	0.3	0.0 (0)	[13]
*Mesocestoides lineatus*	B	9.1	not determined	[14]
*Mesocestoides litteratus*	P	x	not determined	[28]
*Spirometra* spp.	B	9.1	0.0 (0)	[14]
*Spirometra janickii*	P	40	not determined	[12]
*Spirometra erinaceieuropaei*	F	x	not determined	[10]
Trematodes				
*Alaria alata*	P, F	1.7–6	not determined	[12,19]
Heterophyidae **	G	-	8.3 (0–19.3)	Current study
Protozoa				
*Cystoisospora rivolta*/*C. felis* **	P, F, G	0.6–14.7	25 (7.7–42.3)	[13,16]
coccidia	Swi	12.1		[8]
*Toxoplasma gondii*	F, Swe	75.4–86.1 °	not determined	[17,29]
*Toxoplasma/Hammodia* spp. **	G	-	4.2 (0–12.2)	Current study
*Giardia intestinalis* **	G	-	16.7 (1.8–31.6)	Current study
*Cryptosporidium* spp. **	G	-	8.3 (0–19.3)	Current study
*Sarcocystis* spp. **	G	-	29.2 (11–47.4)	Current study

* P: Poland, B: Bosnia–Herzegovina, L: Latvia, F: Finland, E: Estonia, Swi: Switzerland; Swe: Sweden; Sl: Slovakia; G: Germany; ** Parasites detected in current study; CI: confidence interval; x = report of occurrence in individual lynx sample; ° sera prevalence.

**Table 2:** Primer and probe sequences.

Primer	Probe	Orientation	Sequence 5′…3′, (Modifications)	Reference
Gd-80F		Forward	GACGGCTCAGGACAACGGTT	[82]
Gd-127R		Reverse	TTGCCAGCGGTGTCCG	[82]
	Gd-FT		(FAM)-CCCGCGGCGGTCCCTGCTAG-(DDQ1)	[82]
Cp-583F		Forward	CAAATTGATACCGTTTGTCCTTCTG	[82]
Cp-733R		Reverse	GGCATGTCGATTCTAATTCAGCT	[82]
	Cp-TRT		(Texas Red)-TGCCATACATTGTTGTCCTGACAAATTGAAT-(DDQ2)	[82]
Tcanis-F		Forward	CAAATTGATACCGTTTGTCCTTCTG	[84]
Tcati-F		Forward	GCGCCAATTTATGGAATGTGAT	[84]
TCC-R		Reverse	GAGCAAACGACAGCSATTTCTT	[84]
	Tcanis-S		(FAM)-CCATTACCACACCAGCATAGCTCACCGA-(BHQ1)	[84]
	Tcati-S		(Cy5)-TCTTTCGCAACGTGCATTCGGTGA-(BHQ3)	[84]
SarcoFext		Forward	GGTGATTCATAGTAACCGAACG	[85]
SarcoRext		Reverse	GATTTCTCATAAGGTGCAGGAG	[85]
SarcoFint		Forward	CGCAAATTACCCAATCCTGA	[85]
SarcoRint		Reverse	ATCGTCTTCGAGCCCCTAAC	[85]
NC1		Forward	ACGTCTGGTTCAGGGTTGTT	[86]
NC2		Reverse	TTAGTTTCTTTTCCTCCGCT	[86]
TrogloF		Forward	GCACTTGAAATCTTCGACA	[87]
AeluroF		Forward	GCATTTATGCTAGTGATATC	[87]
MetR		Reverse	CCGCTAAATGATATGCTTA	[87]

## Data Availability

The putative *P. pulmonaris*, *C. felis* and *U. stenocephala* sequences obtained from *L. lynx* were deposited in the GenBank database (National Center for Biotechnology Information, HIH, Bethesda, USA) and are available under accession numbers: MZ801778, OL689225 and OK480966.
